# A New Type of Biphasic Calcium Phosphate Cement as a Gentamicin Carrier for Osteomyelitis

**DOI:** 10.1155/2013/801374

**Published:** 2013-04-09

**Authors:** Wen-Yu Su, Yu-Chun Chen, Feng-Huei Lin

**Affiliations:** ^1^Division of Medical Engineering Research, National Health Research Institutes, Miaoli county 350, Taiwan; ^2^Institute of Biomedical Engineering, National Taiwan University, Taipei 100, Taiwan; ^3^Institute of Biomedical Engineering and Material Science, Central Taiwan University of Science and Technology, Taichung 406, Taiwan

## Abstract

Osteomyelitis therapy is a long-term and inconvenient procedure for a patient. Antibiotic-loaded bone cements are both a complementary and alternative treatment option to intravenous antibiotic therapy for the treatment of osteomyelitis. In the current study, the biphasic calcium phosphate cement (CPC), called **α**-TCP/HAP (**α**-tricalcium phosphate/hydroxyapatite) biphasic cement, was prepared as an antibiotics carrier for osteomyelitis. The developed biphasic cement with a microstructure of **α**-TCP surrounding the HAP has a fast setting time which will fulfill the clinical demand. The X-ray diffraction and Fourier transform infrared spectrometry analyses showed the final phase to be HAP, the basic bone mineral, after setting for a period of time. Scanning electron microscopy revealed a porous structure with particle sizes of a few micrometers. The addition of gentamicin in **α**-TCP/HAP would delay the transition of **α**-TCP but would not change the final-phase HAP. The gentamicin-loaded **α**-TCP/HAP supplies high doses of the antibiotic during the initial 24 hours when they are soaked in phosphate buffer solution (PBS). Thereafter, a slower drug release is produced, supplying minimum inhibitory concentration until the end of the experiment (30 days). Studies of growth inhibition of *Staphylococcus aureus* and *Pseudomonas aeruginosa* in culture indicated that gentamicin released after 30 days from **α**-TCP/HAP biphasic cement retained antibacterial activity.

## 1. Introduction

Osteomyelitis is defined as inflammation of bone and marrow cavity [1]. Osteomyelitis causes major morbidity and remains the most feared and difficult infection to treat in orthopedic surgery. In general, it is a protracted commitment to antibiotic therapy for 6~8 weeks. The course of treatment is a long-term and inconvenient procedure for patients. For the treatment of osteomyelitis and prosthesis infection, continuous irrigation and antibiotic-impregnated bone cement beads have become popular because of the resulting higher antibiotic concentration in soft tissue or bone [2, 3].

Various studies have suggested that the local application of antimicrobials clearly provides higher local antibiotic concentrations than those achieved with intravenous application and additionally avoids toxicity with high plasma levels [4, 5]. Antibiotic beads are both a complementary and alternative treatment option to intravenous antibiotic therapy for the treatment of osteomyelitis. Polymethylmethacrylate (PMMA) antibiotic beads were introduced clinically 30 years ago and have been the main local antibiotic delivery system in osteomyelitis therapy until recently [6, 7]. The beads can be impregnated with one or more antibiotics and placed within the wound site at the time of surgery. Ideally, the antibiotics then diffuse out of the beads, maintaining a local concentration above the minimum inhibitory concentration for a period of days or weeks, thereby preventing or diminishing bacterial infection at the site. PMMA beads, however, are associated with some serious disadvantages including uncertain period of antibiotic delivery, as the carrier material is not degradable, and induction of foreign body reaction [8–10].

Recently, much attention has been paid to calcium phosphate cement (CPC) and it has been applied to fractures, filling bone defects, and intensification of bone as bone replacement and augmentation [3, 11]. Calcium phosphate cement, which has a chemical composition close to that of bone, has been extensively explored as bone graft materials [12]. The possibility to use CPCs not only as bone substitutes, but also as carriers for local and controlled release of drugs is very attractive and can be useful in treatments of different skeletal diseases, such as bone tumors, osteoporosis, or osteomyelitis, which normally require long and painful therapies. Several studies have shown that CPC could also be used as a delivery system for therapeutic peptides or antibiotics [13–15]. In general, most of CPCs are composed of one or more calcium phosphates, which upon mixing with a liquid phase, usually water or an aqueous solution, to form a paste, are able to set and harden after being implanted within the body [16–18]. 

In the present study, we developed a biphasic calcium phosphate cement, which consisted of *α*-tricalcium phosphate (*α*-TCP) and hydroxyapatite (HAP). The HAP doped with ammonium hydrogen phosphate [(NH_4_)_2_HPO_4_] could partially convert HAP into *α*-TCP on heating to a temperature up to 1350°C–1450°C. With different concentrations of ammonium hydrogen phosphate addition, we could prepare series of biphasic calcium phosphates in different *α*-TCP/HAP ratios. The prepared *α*-TCP/HAP biphasic cement can set in aqueous environment because the *α*-TCP can be hydrolyzed in water solution and perform segregation. Apatite crystal generated in segregation can interactively set with each other similar to the coagulation of cement. 

*α*-TCP/HAP biphasic cement is a self-setting materials consisting of *α*-TCP and HAP that can harden completely into a uniform microporous hydroxyapatite when mixed in an aqueous solution. By replacing water with an antibiotic solution, *α*-TCP/HAP biphasic cement might be a convenient carrier material for local antibiotic delivery. It might be possible to maintain high concentrations of antibiotics within an infected lesion and allow bone formation in a bone defect at the same time. Since the morbidity of chronic osteomyelitis is due in part to bone loss and the need of multiple surgical procedures, using these antibiotic-loaded CPC in the bone-defect-filling manner may potentially minimize the risk of infection and avoid additional surgical procedures.

For this reason, the aim of this study was to investigate *α*-TCP/HAP biphasic cement as a drug carrier for gentamicin released in the treatment of chronic osteomyelitis. Gentamicin is the primary choice of orthopedic surgeons for local treatment of osteomyelitis [19, 20]. In the current study, the setting properties of gentamicin-loaded *α*-TCP/HAP biphasic cement were evaluated by X-ray diffraction (XRD), fourier transform infrared (FTIR) spectroscopy, and scanning electron microscope (SEM) observation. The release behavior of gentamicin from *α*-TCP/HAP was evaluated over a period of 30 days and the efficacy of the released gentamicin was performed in the germ culture of *staphylococcus aureus* and *pseudomonas aeruginosa*.

## 2. Material and Methods

### 2.1. Preparation of *α*-TCP/HAP Biphasic Cement

*α*-TCP/HAP biphasic cement was prepared by heating a mixture that contains hydroxyapatite (HAP) and ammonium hydrogen phosphate [(NH_4_)_2_HPO_4_]. First, we dissolve 2.19 g of (NH_4_)_2_HPO_4_ powder in 70 mL distilled water uniformly and then add 40 g of HAP powder into the solution. After stirring, the mixture was dried in an oven at 60°C for 72 hours. The dried mixture was crushed with a mortar and ground into fine powder for the following thermal treatment. The powder mixture was heated to 1350°C at a heating rate of 10°C/min and kept at 1350°C for one hour, and then the powder mixture was quenched to room temperature immediately to obtain the *α*-TCP/HAP biphasic cement. 

### 2.2. Preparation and Evaluation of Gentamicin-Loaded *α*-TCP/HAP Biphasic Cement

 Gentamicin sulfate powder was dissolved in distilled water with different concentrations. The gentamicin-loaded *α*-TCP/HAP biphasic cement was prepared by mixing 0.5 g of *α*-TCP/HAP biphasic cement powder and 0.1 mL of gentamicin sulfate solution. The mixed cement pastes were kneaded to a cube with a dimension of 5 mm × 5 mm × 5 mm.

 In the study, we prepared three different groups of gentamicin-loaded *α*-TCP/HAP biphasic cements, which were loaded with various concentrations of gentamicin sulfate (0 wt%, 4 wt% and 16 wt%, resp.) respectively. Each group of gentamicin-loaded *α*-TCP/HAP biphasic cement involved 12 samples, and then the samples were immersed into 10 mL distilled water for 15 min, 2 hr, 4 hr, 8 hr, 16 hr, 24 hr, 48 hr, 72 hr, 96 hr, 120 hr, 144 hr, and 168 hr, respectively. Draw out samples at the end of each period of time, and then immerse them into pure alcohol immediately to stop transferring of *α*-TCP. After 168 hr, all of the samples were analyzed by scanning electron microscope (SEM), X-ray diffraction (XRD), and fourier transform infrared spectroscopy (FTIR).

### 2.3. Antibiotic Release Experiments

 The gentamicin-loaded *α*-TCP/HAP biphasic cements were prepared as described previously; four samples (containing 0, 4, 8, and 16 wt% of gentamicin sulfate) were prepared and then immersed in 10 mL of phosphate buffer solution (PBS) at 37°C incubator, respectively. The PBS was changed and collected at regular time interval. The collected PBS was analyzed by UV-VIS spectrometer to measure the concentration of released gentamicin sulfate.

### 2.4. Bacteria Growth Inhibition by GS Released from *α*-TCP/HAP Biphasic Cements

 In order to evaluate the activity of released gentamicin sulfate, two kinds of bacteria, *Staphylococcus aureus* ATCC 29213 and *Pseudomonas aeruginosa* ATCC 27853, were used to culture with the solution collected from antibiotic release experiments (Section 2.3). The Mueller-Hinton Broth and sterile test tubes (13 × 100 mm) were used in the experiments. First, add bacteria into a test tube that contained 2 mL of Mueller-Hinton Broth, and then adjust the concentration of bacteria to 10^7^/mL, which was used as coordinated bacteria solution for following experiments. After that, 0.1 mL of the coordinated bacteria solution and 0.5 mL of released gentamicin sulfate solution at each time point (obtained from Section 2.3) were added to sterile test tube that contained 2 mL of Mueller-Hinton Broth. Furthermore, test tubes containing broth and bacteria without released gentamicin sulfate solution were used as control groups. All of these test tubes were incubated in an atmosphere containing 5% CO_2_ at 35°C for 18 hours.

## 3. Results

### 3.1. Preparation and Characterization of *α*-TCP/HAP Biphasic Cement

**Figure 1:**
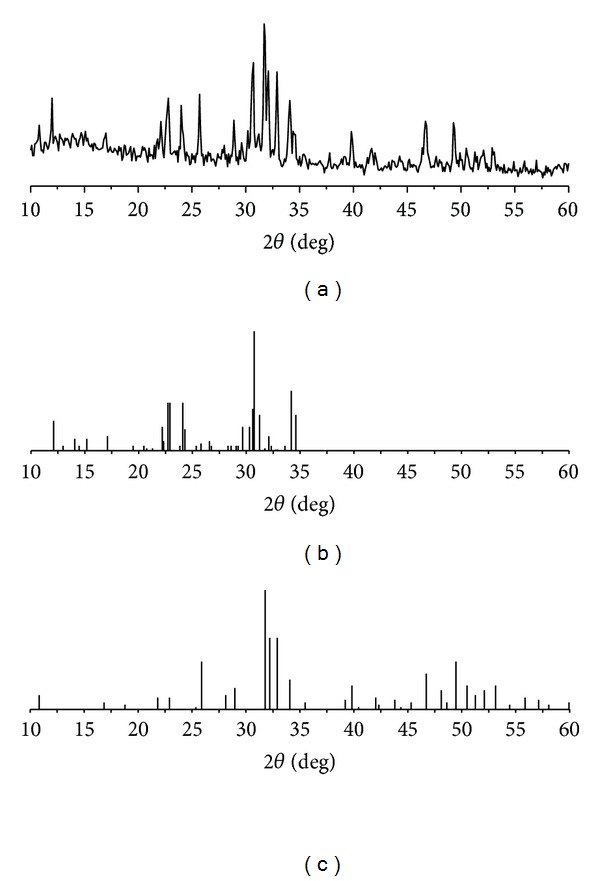
XRD patterns of (a) *α*-TCP/HAP; (b) JCPDS card 9–348 alpha-TCP; (c) JCPDS card 9–432 HAP.

Figure 1(a) shows the XRD patterns of *α*-TCP/HAP biphasic cements. The JCPDS cards 9–348 (Figure 1(b)) and 9–432 (Figure 1(c)) are the standard diffraction pattern of *α*-TCP and HAP, respectively. The major diffraction peak of HAP at 2*θ* = 30.6° and *α*-TCP at 2*θ* = 31.8° could be observed in XRD pattern of *α*-TCP/HAP biphasic cements, which means the end product of thermal treatment consisted of HAP and *α*-TCP.

### 3.2. Preparation and Evaluation of Gentamicin-Loaded *α*-TCP/HAP Biphasic Cement

**Figure 2:**
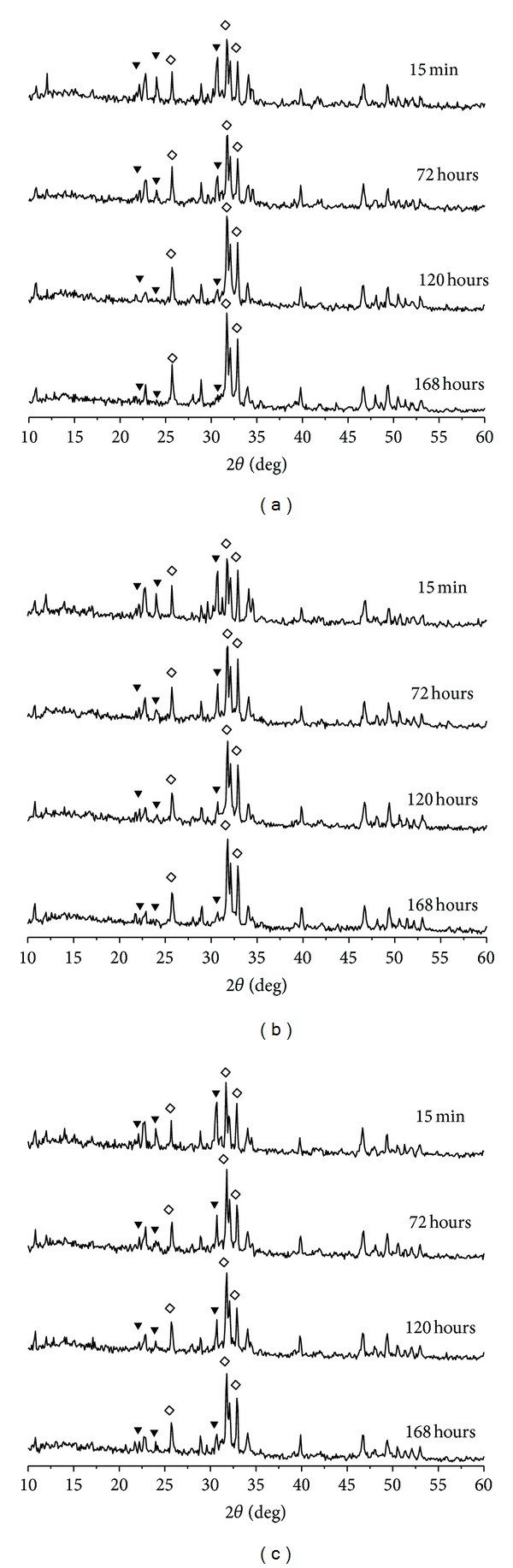
XRD patterns of *α*-TCP/HAP biphasic cement containing (a) 0 wt%; (b) 4 wt%; (c) 16 wt% gentamicin sulfate immersed in distilled water for 15 minutes, 72 hours, 120, and 168 hours. Peak description: (*◊*) HAP; (*▼*) alpha-TCP.

 The effects of adding gentamicin sulfate on the transition of *α*-TCP are shown in Figure 2.  Figure 2(a) shows the XRD patterns of *α*-TCP/HAP biphasic cements without gentamicin sulfate obtained from different setting time points. At *t* = 15 min, the peaks of *α*-TCP and HAP can be observed clearly. As the soaking time increased, the *α*-TCP peak intensities decreased and HAP peaks intensity increased. Finally, the *α*-TCP peaks almost disappear at *t* = 168 hours. Figures 2(b) and 2(c) show that the XRD patterns of *α*-TCP/HAP biphasic cements contained 4% and 16% gentamicin sulfate obtained from different setting time points. The diffraction peaks of *α*-TCP still could be observed even after being immersed in distilled water for 168 hours, which means the phase transition of *α*-TCP is not completed.

**Figure 3:**
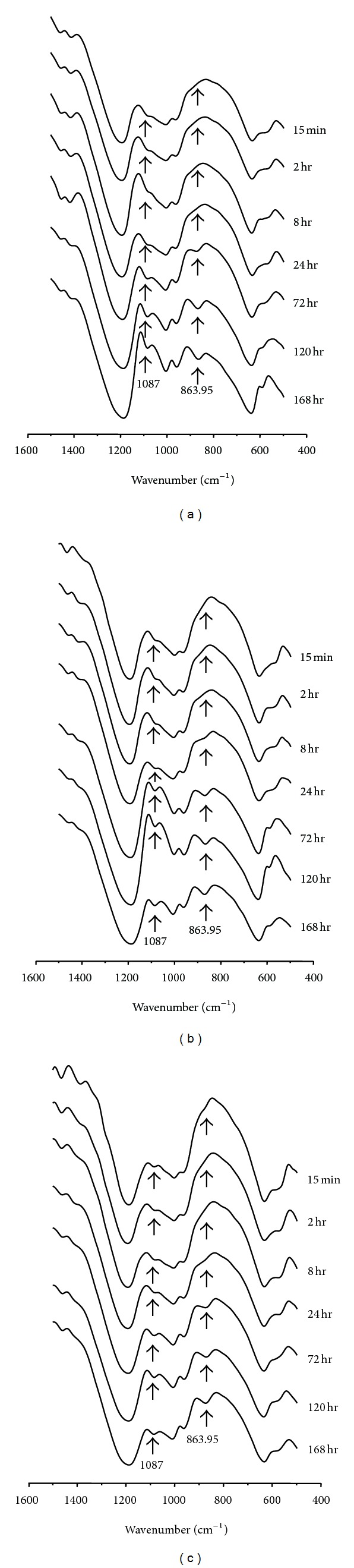
FTIR spectra of *α*-TCP/HAP biphasic cement containing (a) 0 wt%; (b) 4 wt%; (c) 16 wt% gentamicin sulfate immersed in distilled water for 15 minutes, 72 hours, 120, and 168 hours.

 AS we know, *α*-TCP would be gradually converted into apatite in aqueous solution as immersed time increased. However, from the XRD patterns we cannot distinguish HAP from d-HAP. It is known that the deficient HAP has the same crystal structure as HAP, which can be presented by a formula: (d-HAP, Ca_10−*X*_(HPO_4_)_*X*_(PO_4_)_6−*X*_(OH)_2−*X*_). In order to confirm the formation of d-HAP in the CPC system, FTIR analysis was needed.  Figure 3 shows the FTIR spectra of the *α*-TCP/HAP biphasic cements without/with gentamicin sulfate obtained at different setting times. As the soaking time increased, the HPO_4_^−^ band at 865 cm^−1^ was observed and increased in all the spectra of *α*-TCP/HAP biphasic cement with/without gentamicin sulfate; it indicated that the calcium-deficient hydroxyapatite could be formed by the transition of *α*-TCP. 

### 3.3. Antibiotic Release Experiments

**Figure 4:**
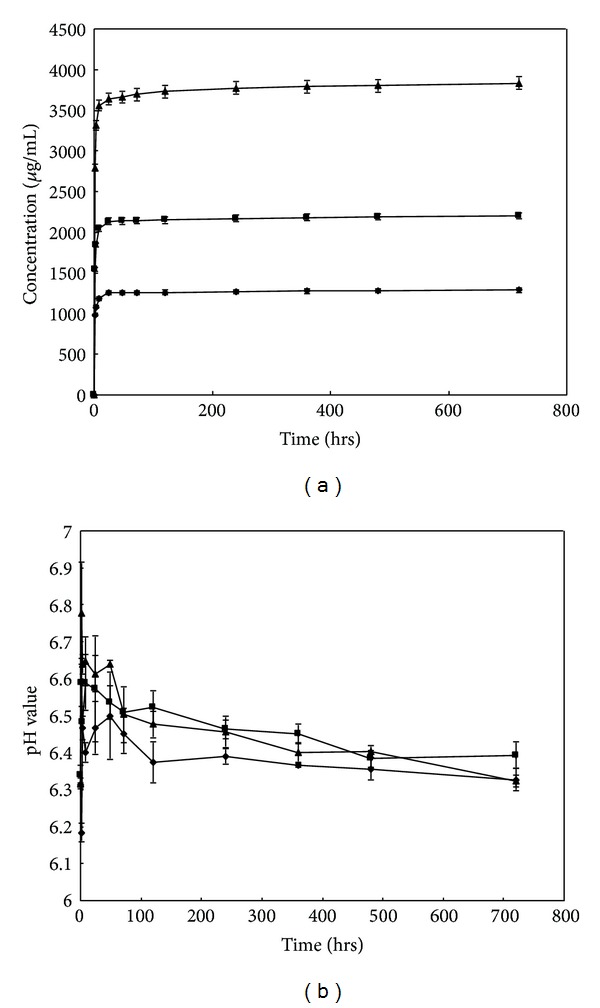
(a) Release curve of gentamicin-loaded *α*-TCP/HAP biphasic cement containing 4 wt% (*◆*), 8 wt% (■), and 16 wt% (▲) of gentamicin sulfate immersed in distilled water for 15 minutes, 72 hours, 120, and 168 hours. (b) pH value of released media of *α*-TCP/HAP biphasic cement containing 4 wt% (*◆*), 8 wt% (■), and 16 wt% (▲) gentamicin sulfate.

Figure 4(a) shows the drug release behavior of *α*-TCP/HAP biphasic cement containing various amounts (4 wt%, 8 wt%, and 16 wt%) of gentamicin sulfate. The drug delivery system showed two-stage release curve. Firstly, gentamicin sulfate was released rapidly from the cement at the initial stage within 24 hours. Secondly, after 24 hours the sustained release of gentamicin sulfate could be observed for 30 days. At the end of release experiments conducted with 4 wt%, 8 wt%, and 16 wt% gentamicin sulfate, the concentrations in the release medium are 7.11 ± 1.01, 12.76 ± 1.03, and 28.76 ± 1.56 *μ*g/mL. The pH value of gentamicin sulfate-released medium was maintained between 6.7 and 6.4 within the release period, as shown in Figure 4(b).

**Figure 5:**
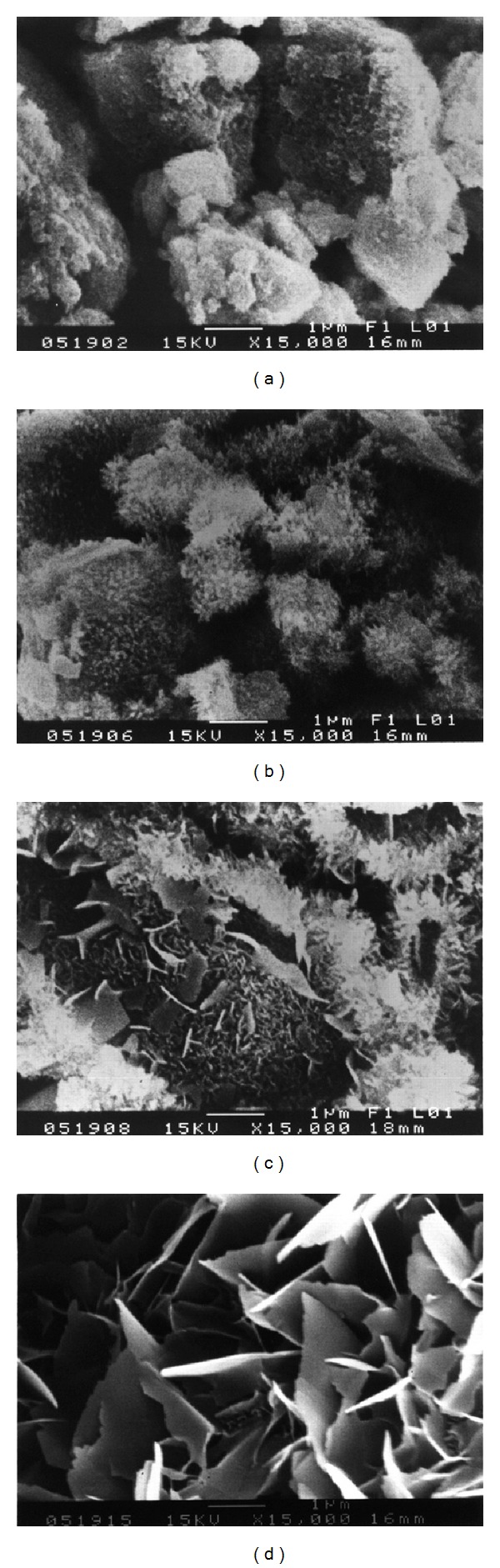
SEM observation of *α*-TCP/HAP biphasic cement immersed in distilled water for a period of time of (a) 15 min; (b) 72 hours; (c) 120 hours; (d) 168 hours.

**Figure 6:**
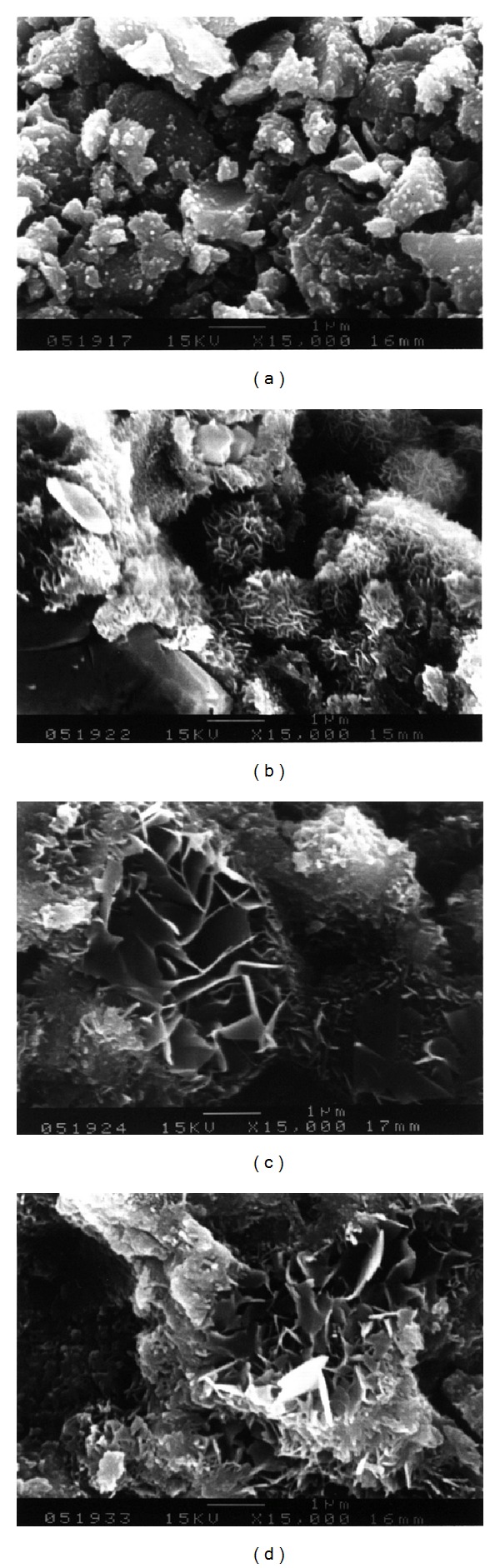
SEM observation of *α*-TCP/HAP biphasic cement containing 4% gentamicin sulfate which was immersed in distilled water for a period of time of (a) 15 min; (b) 72 hours; (c) 120 hours; (d) 168 hours.

**Figure 7:**
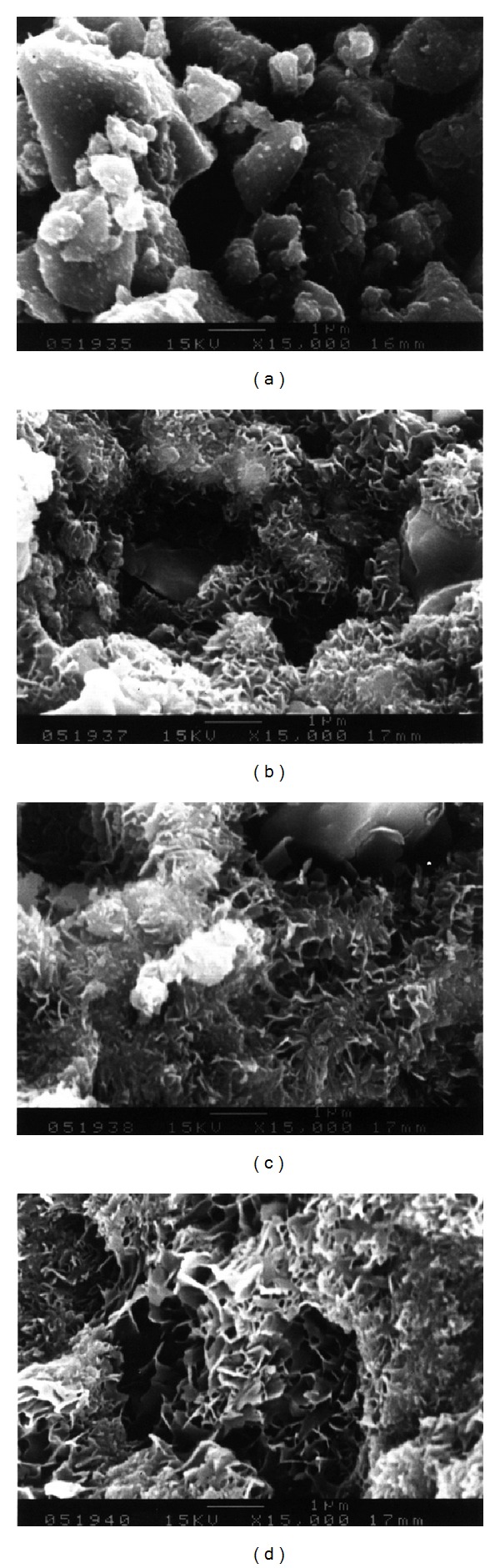
SEM observation of *α*-TCP/HAP biphasic cement containing 16% gentamicin sulfate which was immersed in distilled water for a period of time of (a) 15 min; (b) 72 hours; (c) 120 hours; (d) 168 hours.

 The SEM photos in Figure 5 show the microstructure of the *α*-TCP/HAP biphasic cement without gentamicin sulfate at different setting time points (15 min, 72 hours, 120 hours, and 168 hours). Small needle-like crystals are occasionally observed on the surface of *α*-TCP/HAP particles at early time (15 min). At 72 hours, lots of needle-like crystallites can be observed on the surface of the particles (Figure 5(b)); the crystallites continuously grown and then transformed to plate-like structure after 120 hours, shown in Figure 5(c). Finally, the majority of the *α*-TCP/HAP particles are covered with plate-like of HAP, and area of petal-like crystals significantly increased as the soaking time increased. The microstructures of the cement with different concentrations (4 wt% and 16 wt%) of gentamicin sulfate are showed in Figures 6 and 7, respectively. The similar results were observed when adding various concentration of gentamicin sulfate into cement. However, compared with Figures 5(d), 6(d), and 7(d), the addition of gentamicin sulfate might lead to a thinner and smaller average crystal size.

### 3.4. Bacteria Growth Inhibition by GS Released from *α*-TCP/HAP Biphasic Cements

**Figure 8:**
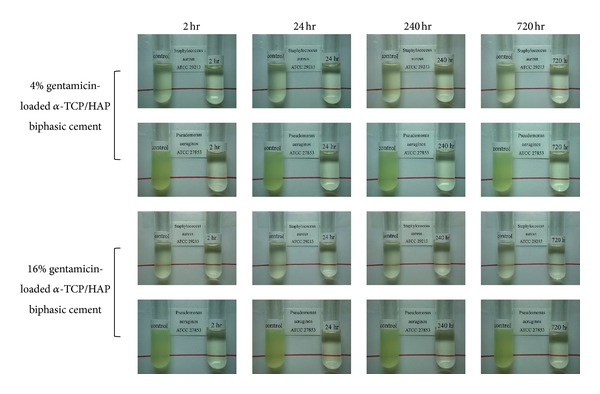
Antibacterial experiments of gentamicin-loaded *α*-TCP/HAP biphasic cement. The left test tube was control group that did not contain released gentamicin sulfate, and the right test tube contained various period of time of released gentamicin sulfate. At the end of release experiments (30 days) conducted with 4 wt%, 8 wt% and 16 wt% gentamicin sulfate, the concentration in the release medium are 7.11 ± 1.01, 12.76 ± 1.03 and 28.76 ± 1.56 *μ*g/mL, respectively. The gentamicin loaded *α*-TCP/HAP biphasic cement can release effective concentration of gentamicin sulfate to inhibit the growth both of *staphylococcus aureus* and *pseudomonas aeruginosa* for 30 days.

Figure 8 shows some representative digital pictures of the released gentamicin sulfate solution cultured with *Staphylococcus aureus* ATCC 29213 and *Pseudomonas aeruginosa* ATCC 27853 in Mueller-Hinton Broth. The released gentamicin sulfate solutions were collected from the previous release experiments and then used for bacteria growth inhibition experiments. At the end of release experiments (30 days) conducted with 4 wt%, 8 wt%, and 16 wt% gentamicin sulfate, the concentrations in the release medium are 7.11 ± 1.01, 12.76 ± 1.03, and 28.76 ± 1.56 *μ*g/mL, respectively. The antibacterial efficacy was evaluated by the clarity of solution; the turbid solution indicated the bacterial growth. In these photos, the left test tube was a control group that did not contain released gentamicin sulfate, and the right test tube contained the released gentamicin sulfate at different time points. It is easy to distinguish the clarity from the line behind test tubes, and it indicated that the released gentamicin sulfate at each time point could inhibit bacteria growth significantly. According to the results of germ culture, the antibiotic-loaded *α*-TCP/HAP biphasic cement could release effective concentration of gentamicin sulfate to inhibit the growth both of *Staphylococcus aureus* and *Pseudomonas aeruginosa* for 30 days.

## 4. Discussion

 When treating osteomyelitis and prosthesis infection, it is often difficult to cure them by antibiotics administered systemically. Usually, osteomyelitis treatment requires prolonged antibiotic therapy, with a course lasting a matter of weeks or months. Further, there is a need of high parenteral dose of antibiotic to achieve effective therapeutic drug concentrations in the infected bone [1]. Therefore, antibiotic-loaded bone cements have become a complementary treatment because of the resulting higher antibiotic concentration in the infected area [2, 5, 8, 20]. Calcium phosphate cement has been used for bone replacement and augmentation because of good biocompatibility and osteoconductivity. There have been some studies about the efficacy and release of antibiotic-impregnated bone cement [21–24]. The present study showed the efficacy of *α*-TCP/HAP biphasic cement as a biocompatible carrier material that allows prolonged release of antibiotics, gentamicin sulfate. Calcium phosphate cements have the significant advantage of setting at ambient temperatures, which means that antibiotics can be incorporated into the matrices during preparation. 

 The significant effect of gentamicin sulfate on the setting properties of the *α*-TCP/HAP biphasic cement is to obstruct the phase transformation of *α*-TCP to HAP. The addition of gentamicin sulfate led to a thinner and smaller precipitate grain size (Figures 5, 6, and 7) because sulfate ions might create more nucleation sites and block the crystal growth [25]. Comparing between Figures 5(a), 6(a), and 7(a), it can be observed that there is a large amount of needle-like d-HAP in Figure 5(a), but it could not be observed that there are conspicuous needle-like precipitates in Figures 6(a) and 7(a). Furthermore, comparing between Figures 5(d), 6(d), and 7(d), the average crystal size in Figure 5(d) was larger than that in Figures 6(d) and 7(d). This phenomenon conjectured that due to the higher concentration of sulfate, the sulfate ions would be the nucleation sites when *α*-TCP hydrolyzed and crystallized in aqueous solution. On the other hand, the large molecular structure of gentamicin sulfate may also be obstacles in the process of grain growth.

 The release curves of different concentrations of gentamicin sulfate in this study are similar in shape, suggesting that the drug release was controlled by diffusion through the interconnected porous structure of the cement. The release behavior of this drug delivery system showed two-stage release; initial stage and sustained stage, respectively. In initial stage, it showed bursting release, the high concentration of released gentamicin sulfate conjectured that due to the adsorbent gentamicin sulfate near the surface of cement. Furthermore, the cement did not form enough needle-like or plate-like structure to block the release of gentamicin in initial stage. So, a large amount of gentamicin sulfate was released in this stage. In fact, bursting release is an advantage for the treatment of osteomyelitis. The effectiveness of antibiotic delivery systems for the local prevention of bacterial infections related to orthopedic implants is strongly dependent on the drug release profile. Since the concentration of bacteria was very high at local in early infection, it is needed to maintain a high local concentration of antibiotic to inhibit bacteria. In sustained stage, the release rate became slower due to the formation of needle-like and plate-like structure. The therapeutic concentration of GS is ≥4 *μ*g/mL for sensitive microorganisms and ≥8 *μ*g/mL for more-resistant microorganisms [26]. At the end of release experiments (30 days) conducted with 4 wt%, 8 wt%, and 16 wt% gentamicin sulfate, the concentrations in the release medium are 7.11 ± 1.01, 12.76 ± 1.03, and 28.76 ± 1.56 *μ*g/mL, respectively. According to the result of our study, the amount of released gentamicin sulfate collected from the three groups (*α*-TCP/HAP biphasic cement containing 4%, 8%, and 16% gentamicin sulfate) in vitro clearly exceeded the minimum inhibitory concentration for *Staphylococcus aureus* and *Pseudomonas aeruginosa*. Many species of organisms, such as *Staphylococcus*, *Enterobacteriaceae,* and *Pseudomonas* species, have been implicated in the etiology of osteomyelitis, with *Staphylococcus aureus* being the most commonly isolated organism. The antibacterial experiments further confirm the activity and efficacy of released gentamicin sulfate at regular time interval (Figure 8).

 Most antibiotics, except for aminoglycosides such as gentamicin and tobramycin, are heat labile. The characteristics of calcium phosphate cement are a high release rate of antibiotics and no heating during the setting process. Many antibiotics have been shown to maintain efficacy when mixed with bone cement [11, 14, 15, 21–24]. The requirements of such antibiotics are that they are heat stable and hydrophilic. Release of the antibiotic in such bone cement systems depends on the rate of dissolution of drug in its matrix allowing its penetration through the porous structure of the carrier. The most commonly used antibiotics include gentamicin, tobramycin, and vancomycin. Gentamicin remains the most effective antibiotic to incorporate with bone cement due to its high solubility, heat stability, and bactericidal activity at low concentration [20]. Vancomycin and tobramycin are both water-soluble and available in powder form. Mixing of more than one antibiotic into bone cement has been shown to have a synergistic effect [27, 28]. The combination of different antibiotics can increase the antimicrobial spectrum, and also it would potentially lead to increased concentrations of antibiotics in the local area. The combination of antibiotics is supposed not only to be more powerful, but also to prevent the emergency of resistant strains through the synergistic action of two antibiotics at the same time [29]. 

 Prevention of osteomyelitis and its treatment are still a challenge not only to the veterinary orthopedic surgery but also to the human subjects. Low systemic toxicity and high local antibiotic concentrations are the major advantages of local antibiotic delivery system. Furthermore, there is a possibility that CPC allows bone formation in a bone defect caused by infection because CPC has a character of bone replacement and augmentation. 

## 5. Conclusion

 The use of *α*-TCP/HAP biphasic cement as a drug delivery system for gentamicin sulfate appears to be very promising because gentamicin sulfate could be sustainably released for at least 30 days and the biological activity of gentamicin sulfate was preserved when mixed with the *α*-TCP/HAP biphasic cement. For clinical application, gentamicin-loaded *α*-TCP/HAP biphasic cement may be able to maintain a high local concentration of gentamicin sulfate, which was beneficial to the treatment of osteomyelitis or prosthesis infection.
